# Systematic characterization of the components and molecular mechanisms of Jinshui Huanxian granules using UPLC-Orbitrap Fusion MS integrated with network pharmacology

**DOI:** 10.1038/s41598-022-16711-4

**Published:** 2022-07-21

**Authors:** Jie Yuan, Di Zhao, Xue-Fang Liu, Yan-Ge Tian, Hao-Jie Zhang, Su-Xiang Feng, Jian-Sheng Li

**Affiliations:** 1 College of Pharmacy, Henan University of Chinese Medicine, Zhengzhou, Henan China; 2 Collaborative Innovation Center for Chinese Medicine and Respiratory Diseases Co-Constructed by Henan Province & Education Ministry of P.R. China, Zhengzhou, Henan China; 3 Academy of Chinese Medicine Sciences, Henan University of Chinese Medicine, Zhengzhou, Henan China; 4 The First Affiliated Hospital, Henan University of Chinese Medicine, Zhengzhou, Henan China

**Keywords:** Plant sciences, Diseases, Medical research, Pathogenesis

## Abstract

Jinshui Huanxian granules (JSHX) is a clinical Chinese medicine formula used for treating pulmonary fibrosis (PF). However, the effective components and molecular mechanisms of JSHX are still unclear. In this study, a combination approach using ultra-high performance liquid chromatography-Orbitrap Fusion mass spectrometry (UPLC-Orbitrap Fusion MS) integrated with network pharmacology was followed to identify the components of JSHX and the underlying molecular mechanisms against PF. UPLC-Orbitrap Fusion MS was used to identify the components present in JSHX. On the basis of the identified components, we performed target prediction using the SwissTargetPrediction database, protein–protein interaction (PPI) analysis using STRING database, and Gene Ontology (GO) and Kyoto Encyclopedia of Genes and Genomes (KEGG) pathway enrichment analysis using Metascape and constructed a component-target-pathway network using Cytoscape 3.7.2. Molecular docking technology was used to verify the affinity between the core components and targets. Finally, the pharmacological activities of three potentially bioactive components were validated in transforming growth factor β1 (TGF-β1)-induced A549 cell fibrosis model. As a result, we identified 266 components, including 56 flavonoids, 52 saponins, 31 alkaloids, 10 coumarins, 12 terpenoids and 105 other components. Of these, 90 validated components were predicted to act on 172 PF-related targets and they exhibited therapeutic effects against PF via regulation of cell migration, regulation of the mitogen-activated protein kinase (MAPK) cascade, reduction of oxidative stress, and anti-inflammatory activity. Molecular docking showed that the core components could spontaneously bind to receptor proteins with a strong binding force. In vitro, compared to model group, hesperetin, ruscogenin and liquiritin significantly inhibited the increase of α-smooth muscle actin (α-SMA) and fibronectin (FN) and the decrease of e-cadherin (E-cad) in TGF-β1-induced A549 cells. This study is the first to show, using UPLC-Orbitrap Fusion MS combined with network pharmacology and experimental validation, that JSHX might exert therapeutic actions against PF by suppressing the expression of key factors in PF. The findings provide a deeper understanding of the chemical profiling and pharmacological activities of JSHX and a reference for further scientific research and clinical use of JSHX in PF treatment.

## Introduction

Pulmonary fibrosis (PF) is a chronic, progressive and fatal lung disease, characterized by irreversible lung structure damage and exuberant extracellular matrix protein deposition^1,2^. The main clinical symptoms are dry cough and progressive dyspnea^3^. Accumulating evidence shows that the important pathogenic mechanisms that promote PF, mainly involve damage of the alveolar histological structure, proliferation of fibroblasts and myofibroblasts, further leading to the deposition of extracellular matrix and lung structural remodeling^4^. At the molecular mechanism level, oxidative stress is one of the important mechanisms underlying PF, which mainly causes the oxidation-antioxidation imbalance generation by mitochondria and the enzyme system^5,6^. Oxidative stress affects the normal structure and physiological function of the lungs by inducing inflammation and regulating fibrosis-related cellular signaling pathways, such as mitogen-activated protein kinase (MAPK), nuclear factor kappa B (NF-κB), phosphatidylinositol-3-kinase/protein kinase B (PI3K/Akt), etc.^7^. At present, glucocorticoids, immune suppressants, pirfenidone (PFD) and nintedanib, are mainly used to treat PF^8,9^. However, their curative effect is limited or there are side effects^10^. Thus, it is necessary to find alternative drugs for PF treatment. Chinese medicine (CM) has exerted multi-target effects on PF and regulated oxidant stress, reduced the expression of inflammatory factors, and induced apoptosis of pulmonary fibroblasts, etc.^11,12^.

Jinshui Huanxian granules (JSHX), a clinical Chinese medicine formula, had been widely used for treating PF^13,14^, which is composed of 10 herbs, including Panax ginseng C. A. Mey. (PG), Ophiopogonis Radix (OR), Radix Rehmanniae Praeparata (RRP), Fritillariae Thunbergii Bulbus (FTB), Trichosanthis Fructus (TF), Moutan Cortex (MC), Epimedii Folium (EF), Ginkgo Semen (GS), Citri Reticulatae Pericarpium (CRP) and Glycyrrhizae radix et rhizome (GR). Our previous studies showed JSHX can effectively improve lung function, reduce lung tissue injury and collagen deposition in PF rats^15^. A controlled trial revealed that JSHX significantly improve the inflammatory response and oxidative stress of lung injury caused by PM2.5 exposure compared with placebo^16^. Moreover, pharmacodynamic study clarified that JSHX could suppress the TGF-β1-induced differentiation of fibroblasts into myofibroblasts through reducing the oxidative response by upregulating Nrf2 signaling in PF rats^17^. However, little research has been done to elucidate the comprehensive material basis and the molecular mechanisms of JSHX, further studies are urgently needed to clarify the bioactive components in JSHX, and further clarify its medicinal and clinical application values. Chinese Medicine formula has complex components and diverse targets. We hypothesized that the bioactive components in JSHX play a role in PF treatment through the molecular mechanism of anti-inflammatory, antioxidant, inhibition of alveolar epithelial stromal and fibroblast activation.

**Figure 1:**
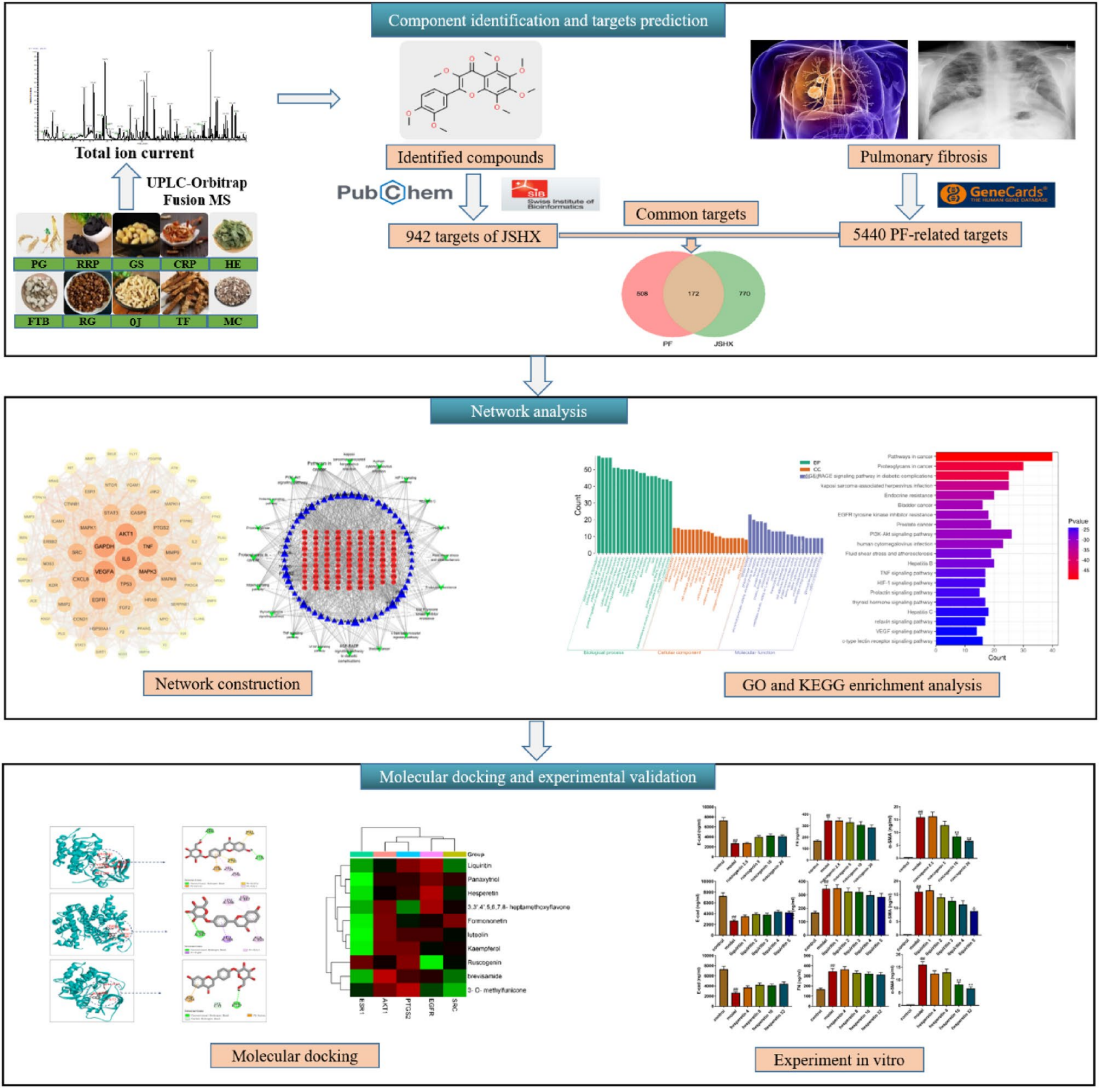
The whole framework based on an integration strategy of UPLC-Orbitrap Fusion MS integrated with network pharmacology, molecular docking and experiment in vitro.

Thus, in this study, we developed an integrative approach to clarify the effect of JSHX on PF by combining UPLC-Orbitrap Fusion MS analysis and network pharmacology. UPLC-Orbitrap Fusion MS technology was performed to characterize the chemical profile of JSHX. Network pharmacology and molecular docking were used to conduct data mining on its chemical components to explore the bioactive components, potential targets, biological processes and signaling pathways relevant to PF treatment, and the effects of bioactive components were further validated in TGF-β1-induced A549 cells. The protocol is shown in Fig. 1.

## Materials and methods

### Materials and reagents

HPLC-grade methanol and acetonitrile were obtained from TEDIA (Fairfield, OH, USA), MS-grade formic acid was purchased from Thermo Fisher Scientific (Waltham, USA). Ultra-pure water was prepared by a Milli-Q water purification system (Millipore, Merk, USA). All other chemicals and reagents were analytical grade. Panax ginseng C. A. Mey. (PG), Ophiopogonis Radix (OR), Radix Rehmanniae Praeparata (RRP), Fritillariae Thunbergii Bulbus (FTB), Trichosanthis Fructus (TF), Moutan Cortex (MC), Epimedii Folium (EF), Ginkgo Semen (GS), Citri Reticulatae Pericarpium (CRP) and Glycyrrhizae radix et rhizome (GR) were purchased from Ruilong Pharmaceutical Co., LTD (Zhengzhou, China), Standard Basis: *Chinese Pharmacopoeia 2020 edition.* These herbs were identified by Prof. Sui-Qing Chen, Henan University of Chinese Medicine, Zhengzhou, China. The voucher specimen was stored at Scientific Research Center, Henan University of Chinese Medicine, Zhengzhou, China.

As reference standards, 37 pure components were used. Seventeen reference standards, including Rehmannioside D (CHB180129), Ferulic Acid (CHB180206), Ginsenoside Rg1 (CHB190110), Neocnidilide (CHB1810707), Isocorynoline (CHB181012), Ginsenoside Rg2 (CHB190111), Hesperetin (CHB180524), Liquiritin (CHB180608), Cynaroside (CHB180111), Ginsenoside Rc (CHB190104), Ginsenoside Rf (CHB190108), Astragalin (CHB181115), Ginsenoside Rb2 (CHB131014), Ginsenoside Re (CHB170926), Tangeritin (CHB150806), Catalpol (CHB181129), Apigenin-7-O-Glucoside (CHB161226) were purchased from Chengdu Cloma Biological Technology Co., LTD (Chengdu, China); Eleven reference standards, including Peimisine (must-19072508), Epimedin A (must-11062311), Epimedin B (must-11062312), Ginkgolide B (must-17073101), Hesperidin (must-16041806), Nobiletin (must-16070901), Paeonol (must-16071405), Ruscogenin (must-15021807), luteolin (must-14050411), Isoacteoside (must-19103104), Echinocystic acid (PS16091901) were all purchased from Chengdu Mansite Biotechnology Co., Ltd (Chengdu, China); Wedelolactone (PJ0629RA13) and Epimedin C (20,120,118) were purchased from Shanghai Yuanye Biological Technology Co., LTD (Shanghai, China); Seven reference standards, including Ginsenoside Rb1 (110,704–201,726), Baohuoside I (111,852–201,102), Peimine (110,750–201,612), Peiminine (110,751–201,712), Icariin (110,737–201,516), Rutinum (100,080–201,408), Quercitrin (111,538–201,105) were purchased from China Food and Drug Testing Institute (Beijing, China). The purity of each component was ≥ 98% by HPLC analysis.

Roswell Park Memorial Institute (RPMI) 1640 medium, Radio Immunoprecipitation Assay (RIPA) Lysis Buffer, Thiazolyl Blue Tetrazolium Bromide (MTT) and trypsin–EDTA were obtained from Beijing Solarbio Science & Technology Co., Ltd (Beijing, China), Fetal bovine serum (FBS) was purchased from Zhejiang Tianhang Biotechnology Co., Ltd. (Zhejiang, China), Enzyme-linked immunosorbent assay (ELISA) kits for E-cad, α-SMA, and FN were obtained from BOSTER Biological Technology co., ltd (Wuhan, China).

### Qualitative analysis of JSHX

#### Sample preparation

Five herbs (PG, RRP, OR, TF, GR) were decocted twice with 12 times the amount of water for 1 h and filtered, and the filtrate was concentrated and reserved. Five herbs (FTB, MC, EF, GS, CRP) were weighed and added in a round-bottom flask with 10 times the amount of 70% ethanol, reflux-heated for 1 h twice and filtered. The filtrate was concentrated and ethanol removed, and this filtrate was then combined with filtrate 1. The mixture was concentrated to thick paste with a relative density of 1.18–1.22, dried at 60 °C under reduced pressure, and crushed into a fine powder. An appropriate amount of dextrin was added and mixed well, 80% ethanol was used as a wetting agent to make grains, and the grains were dried at 60 °C to obtain JSHX. The plants research in this study complies with the *Chinese Pharmacopoeia 2020 edition*.

JSHX (2 g) was accurately weighted and added in a 100 mL round-bottom flask with 25 mL of methanol, the mixture was weighed, reflux-heated for 1 h, cooled to room temperature, weighed again to make up for the lost weight, and filtered, the sample solutions were obtained.

#### Standard solution preparation

Accurately weigh an appropriate amount of each reference standards into a 10 mL volumetric flask, methanol was added to dissolve and dilute to the mark, single-component reference stock solutions were obtained and stored at 4 °C before use. Then, the appropriate amount of stock solution was added to a 25-ml volumetric flask, and methanol was added to reach the volumetric mark, the mixed reference solution was obtained. The solutions were filtered through 0.22 μm microporous membranes and stored at 4 °C before use.

#### Chromatographic and mass spectrometric conditions

An Accucore^TM^C_18_ column (2.1 mm × 100 mm, 2.6 μm) was applied with a constant flow rate of 0.2 mL/min at 30 °C. The mobile phase consisted of methanol (A) and 0.1% formic acid (B) with the following gradient elution: 0–5 min (95–79% B), 5–20 min (79–55% B), 20–41 min (55–25% B), 41–48 min (25–14% B), 48–58 min (14–5% B), 58–60 min (5–0% B). The injection volume was 5 μL.

High-resolution MS detection and analysis were performed on an UPLC-Orbitrap Fusion MS (Thermo Scientific) equipped with an ESI source. The acquisition parameters were set as follows: Vaporizer temperature: 275 °C; Ion Trasfer Tube temperature: 300 °C; Carrier gas (N2); Sheath gas flow rate is 35 arb; Aux gas flow rate is 5 arb; the spray voltage was set at 3.5 (positive ion mode) and 2.5 (negative ion mode) kV; the collision energy was set at 35–55 eV. The sample was analyzed in both positive and negative ion Full MS/dd-MS2 modes with the first-level full scan (resolution: 50,000) and the second-level scan (resolution: 60,000), and the mass range was recorded from m/z 120–1200.

#### Identification of components

JSHX was identified by the optimized UPLC-Orbitrap Fusion MS method. The possible chemical composition (with an error of less than 5 ppm) was determined using Xcalibur based on map data, precise molecular weight and resulting fragment ions. The data was analyzed using Compound Discoverer software to integrate ion peak information, attribution information, ChemSpider, mzCloud, and other databases of characteristic fragments integrated with existing chemical composition information reports. The structure of components was inferred using the cracking prediction of MassFrontier and its cracking rule.

### Network pharmacology

#### Target prediction

The structures of validated components present in JSHX were generated using the PubChem database (https://pubchem.ncbi.nlm.nih.gov/) and saved in SDF format. These SDF documents were uploaded to the SwissTargetPrediction database (http://www.swisstargetprediction.ch/) for target prediction. For more accurate prediction of the target gene of each component, relevant parameters were set (probability ≥ 0.1). Furthermore, the biological targets related to PF were selected from the GeneCards database (https://www.genecards.org/) using “Pulmonary Fibrosis” as the keywords. Then, the intersection of the predicted targets from JSHX and the biological targets of PF was taken and the overlapping targets were screened out as potential targets.

#### Construction of PPI network

The potential targets identified were added to the STRING database. The screening condition used was “Homo sapiens,” and the other parameters were set by default. Cytoscape (version 3.7.2; https://cytoscape.org/) was used to construct a protein–protein interaction (PPI) network. A network analyzer in Cytoscape was used to analyze relevant topological parameters. Using three parameters, degree, betweenness centrality (Bc), and closeness centrality (Cc), a topology analysis of the PPI network was performed to determine hub genes for further analysis.

#### GO and KEGG pathway enrichment analyses

The potential targets identified were uploaded into the Metascape database (https://metascape.org/gp/index.html) for GO and KEGG pathway enrichment analysis to obtain the information of pathways^18–20^. The Bioinformatics Data analysis and Visualization online platform (http://www.bio-informatics.com.cn/) was adopted to perform GO and KEGG pathway analysis and visualize the bar chart in this study. During this procedure, the significance level was set as p ≤ 0.01, and the organism was selected as “Homo sapiens.”

#### Component-target-pathway network construction

To further clarify the relationship among components, targets and pathways, a component-target-pathway network was constructed using Cytoscape 3.7.2. The core components nodes were obtained based on the three parameters: degree, Bc and Cc.

#### Molecular docking

Molecular docking technology was used to verify the affinity between the core components and targets. The 3D structure of the core targets in the first five degrees of JSHX in PF treatment was downloaded from the RCSB PDB database (https://www.rcsb.org/). Pymol (version 1.8; https://pymol.org/2/) software was used to remove water molecules and separate the primary ligand. After saving, the structure was imported into Autodock Tools 1.5.6 and saved in “pdbqt” format. Chem 3D (version 19.0.0.22; https://www.3dchem.com/index.html) software was used to download the mol2 files of the top 10 core components, and they were imported into Autodock Tools (version 1.5.6; https://autodock.scripps.edu/) and saved in pdbqt format. Finally, docking was carried out using Autodock vina (version 1.1.2; https://vina.scripps.edu/). Discovery Studio Client (version 4.5; https://www.3ds.com/products-services/biovia/products/molecular-modeling-simulation/biovia-discovery-studio/) was used to visualize docking results and establish a docking interaction model diagram.

### Experimental validation in vitro

#### Cell culture

A549 cells were obtained from Cell Resource Center of Shanghai Academy of Biological Sciences, Chinese Academy of Sciences (Shanghai, China). Cells were cultured in RPMI 1640 medium supplemented with 10% FBS and maintained in a humidified incubator with 5% CO_2_ at 37 °C. When the cells were grown to 80–90%, they were digested with 0.25% trypsin–EDTA, collected, subcultured or tested.

#### Experimental grouping

Ruscogenin, liquiritin and hesperetin were dissolved by DMSO and diluted to appropriate concentrations before use, respectively. The cells were divided into model group: A549 cells were stimulated in complete culture medium containing TGF-β1 (7.5 ng/ml) and 10% FBS; Control group: A549 cells were cultured in complete medium supplemented with 10% FBS. Dosage group: on the basis of the model group, the dosing groups included hesperetin, ruscogenin and liquiritin.

#### Cell viability assay

The cell viability was measured with MTT assay. A549 cells (1 × 10^4^ cells/well) were planted into a 96-well plate, and incubated at 37 °C with 5% CO_2_ for 24 h. Then the cells were treated with PBS or different dosages of hesperetin (2, 4, 8, 16, and 32 μg/ml), liquiritin (1, 2, 3, 4, and 5 μg/ml), and ruscogenin (2.5, 5, 10, 20, and 40 μg/ml). After incubation for 24 h, 10 µl 0.5 mg/ml MTT was added to each well, and incubated for 4 h. The cell culture was replaced with DMSO (100 µl/well). The absorbance was measured at 570 nm by using a microplate reader.

#### E-cad, α-SMA, FN expression

A549 cells (2 × 10^6^ cells/plate) were incubated in 6-well plates for 24 h, then treated with hesperetin (4, 8, 16, 32 μg/ml), ruscogenin (2.5, 5, 10, 20 μg/ml) and liquiritin (1, 2, 3, 4, 5 μg/ml), and incubated at 37 °C with 5% CO_2_ in a humidified atmosphere for 48 h. Supernatants were collected, centrifuged and stored at − 80 °C for testing. RIPA Lysis Buffer 300 μl/well was added to lysate cells. 10 min later, the lysate was collected, the supernatant was collected by centrifugation, and levels of E-cad, α-SMA, FN in the supernatant were determined by ELISA.

#### Statistical analysis

Data were expressed as mean ± standard deviation (SD). Statistical Differences in multiple groups were performed by analysis of variance (ANOVA). *P*-values < 0.05 was considered statistically significant.

## Results and discussions

### Identification of components of JSHX

**Figure 2:**
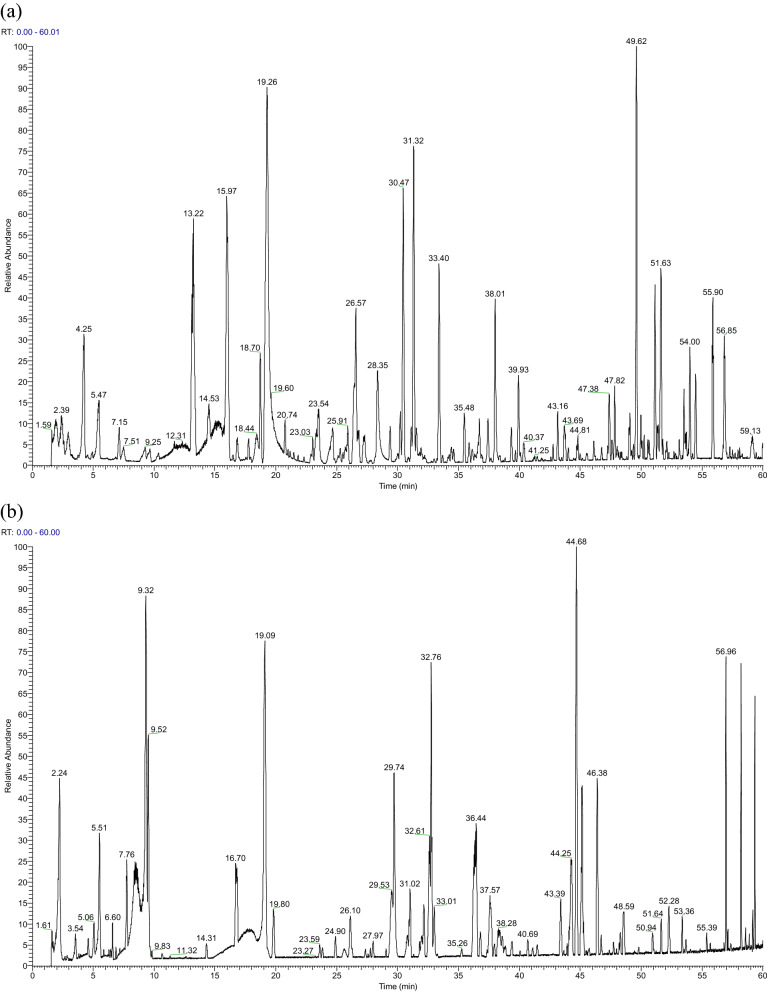
The total ion chromatograms (TICs) of JSHX. (**a**) TIC of JSHX in positive ion mode. (**b**) TIC of JSHX in negative mode.

A total of 266 components were identified in JSHX: 56 flavonoids, 52 saponins, 31 alkaloids, 10 coumarins, 12 terpenoids and 105 other components including quinones, esters, and organic acids, etc. Of the 266 components, 37 components were unambiguously identified via comparison with reference standards. The primary and secondary information was compared with the database for confirmation. The total ion chromatograms (TICs) of positive and negative ions were shown in Fig. 2, and detailed information about the 266 peaks identified was listed in Supplementary Table S1. As the identified components were mainly flavonoids, saponins and alkaloids, their characteristics were summarized.

#### Flavonoids

Flavonoids mainly originate from EF, CRP, GS in JSHX, a total of 56 flavonoids were identified, including 7 flavone aglycones and 49 flavone O-glycosides. Flavone aglycones usually undergo Retro-Diels-Adel (RDA) reaction and lose small-molecule fragments of H_2_O, CO, CO_2_, and CHO through collision-induced dissociation. In flavone glycosides, glycosidic bonds are easily broken, and they can lose glycosyl groups to generate aglycon ions^21^.

**Figure 3:**
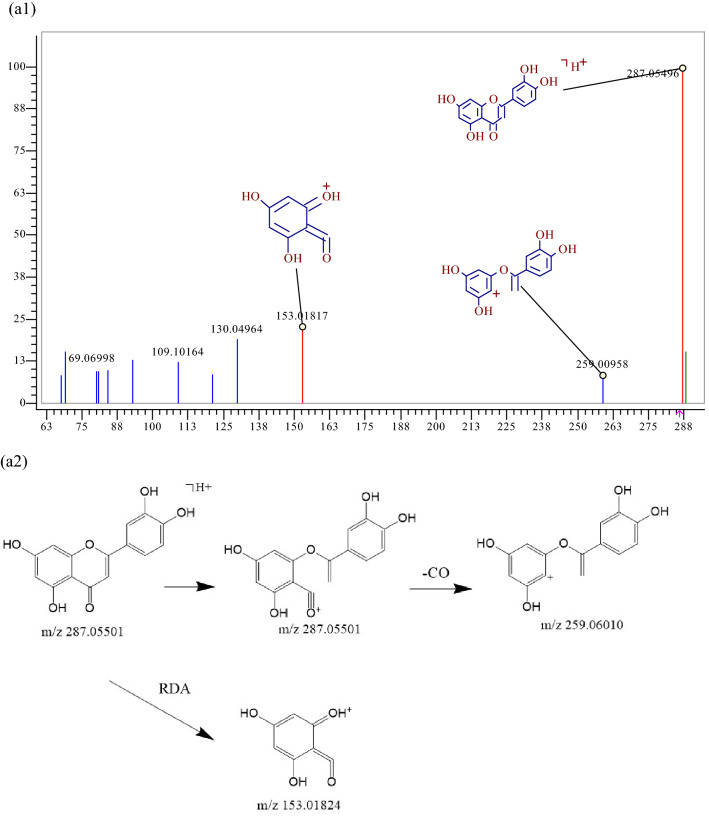

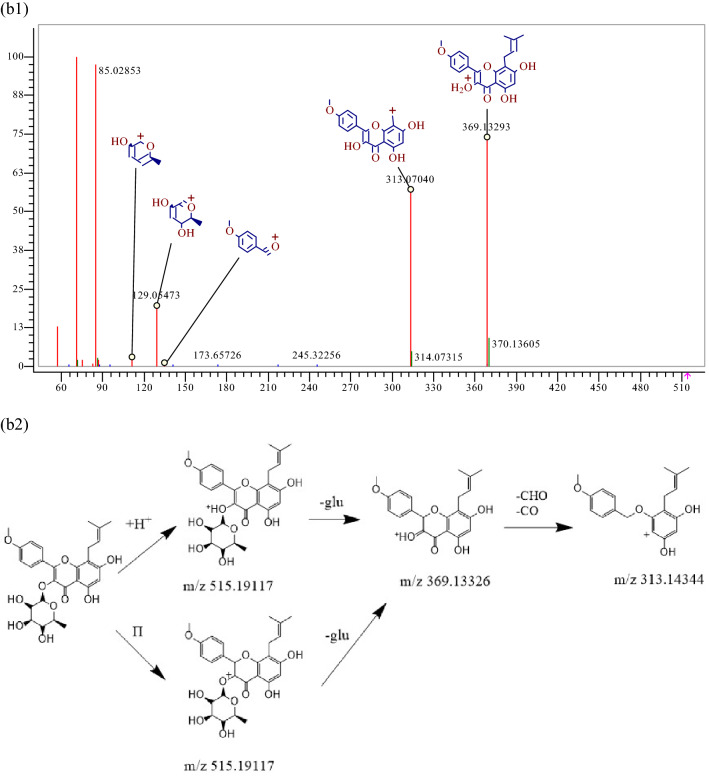
MS^2^ spectra and cracking diagram of (**a**) luteolin (**b**) baohuoside I.

Aglycones 177 possessed quasi-molecular ions [M+H] + at m/z 287.05496, and the chemical composition was C_39_H_50_O_20_. During collision-induced dissociation, the RDA reaction occurs and fragment ions of m/z153.01817 are generated. Small molecule fragments are also lost: a CO molecule were lost to generate a fragment ion [M+H−CO] + at m/z 259.00958, as shown in Fig. 3. Similarly, components 59, 65, 123, 125, 143, and 155 were confirmed as naringenin, quercetin, kaempferol, baicalein, apigenin, and 3,5,7-Trihydroxy-2-(4-hydroxyphenyl)-8-(3-methylbut-2-enyl)-2,3-, respectively. Flavone glycosides 203 possessed quasi-molecular ions [M+H]+ at m/z 403.13874, and the chemical composition is C_27_H_30_O_10_. A glucosyl group is lost, generating a fragment ion [M+H−glu]  + at m/z 369.13293. On this basis, the B-ring is broken, and a CHO molecule and a CO molecule were lost to generate a fragment ion [M+H−CHO−CO] + at m/z 313.14344, as shown in Fig. 3, and component 203 was further identified as baohuoside I by reference standard. Similarly, constituents 136, 137, 140, and 141 were confirmed as epimedin A, epimedin B, icariin and epimedin C via reference standards, respectively.

During MS information scanning, flavonoids mainly underwent deglycosylation and dehydration, characterized by the loss of glucose (162), rhamnose (146), xylose (132) and (18) H_2_O, and finally formed a structure with m/z 369 as the parent nucleus. Connecting different sugar groups produces corresponding secondary fragments, and m/z 369 may break into m/z 313, m/z 515 break into m/z 129, and m/z 129 loses H_2_O to get component 111, which can assist in analysis. According to these rules, 56 flavonoids were identified, including components 35, 37, 43, 50, 51, 52, 58, 59, 65, 66, 69, 73, 74, 76, 77, 79, 80, 81, 83, 88, 96, 97, 104, 107, 111, 112, 114, 115, 123, 125, 131, 135, 136, 137, 138, 140, 141, 142, 143, 146, 151, 152,153, 154, 155, 159, 164, 165, 167, 170, 171, 173, 176, 177, 185, 203.

#### Saponins

Saponins mainly originate from PG, OR in JSHX, include triterpenoid saponins and steroidal saponins^22^. Triterpenoid saponins can be divided into three categories according to structure: ginsenoside diol (A), ginsenoside triol (B), and oleanolic acid (C). In saponins, glycosidic bonds are easily broken and they can lose glycosyl groups to generate aglycon ions. By removing multiple glycosyl groups-glu (162), rha (146), and ara (132) to form characteristic ions, A, B and C finally form the characteristic parent nucleus at m/z 459.3843, m/z 459.3792 and m/z 455.3530, respectively^23^.

Triterpenoid saponins 197 possessed quasi-molecular ions [M−H]− at m/z 1107.59566, and the chemical composition was C_54_H_92_O_23_. During collision-induced dissociation, a glucose group was lost in turn to generate fragment ion [M−H−glu]− at m/z 945.54291, [M−H−2glu]− at m/z 783.49017, [M−H−3glu]− at m/z 621.4358, [M−H−4glu]− at m/z 459.3841, respectively. The parent nucleus of m/z 459 is the structural parent nucleus of Ginsenoside diol, and component 197 was further identified as Ginsenoside Rb1 by reference standard. Due to the presence of formic acid in the mobile phase, an [HCOO]– adjoint ion peak may be generated during cleavage. For example, the excimer ion peak of component 199 at RT = 36.92 min was m/z 945.54284 [M−H]−, and its chemical composition was C_48_H_82_O_18_, which is presumed to be Ginsenoside Re or Ginsenoside Rd. M/z 991 is found in the spectrum, it is presumed that it is an additive ion peak [M−H+HCOO]−, and the abundance of molecular ion peak of m/z 945.54284 [M-H]- is relatively high. At RT = 39.62, m/z 991.54761 and m/z 945.54234 are also found, which are presumed to be [M−H++HCOO]−, [M−H]− respectively. Component 199 is identified as Ginsenoside Re or Ginsenoside Rd. Since Ginsenoside Re and Ginsenoside Rd are isomers, after comparison with the reference standard, component 199 was Ginsenoside Re, RT = 36.92 min, namely component 214 was Ginsenoside Rd, RT = 39.62. Similarly, components 126, 129, 198, 208, and 209 were confirmed as Ginsenoside Rg1, Rf, Rg2, Rb2, and Rc via reference standards, respectively.

**Figure 4:**
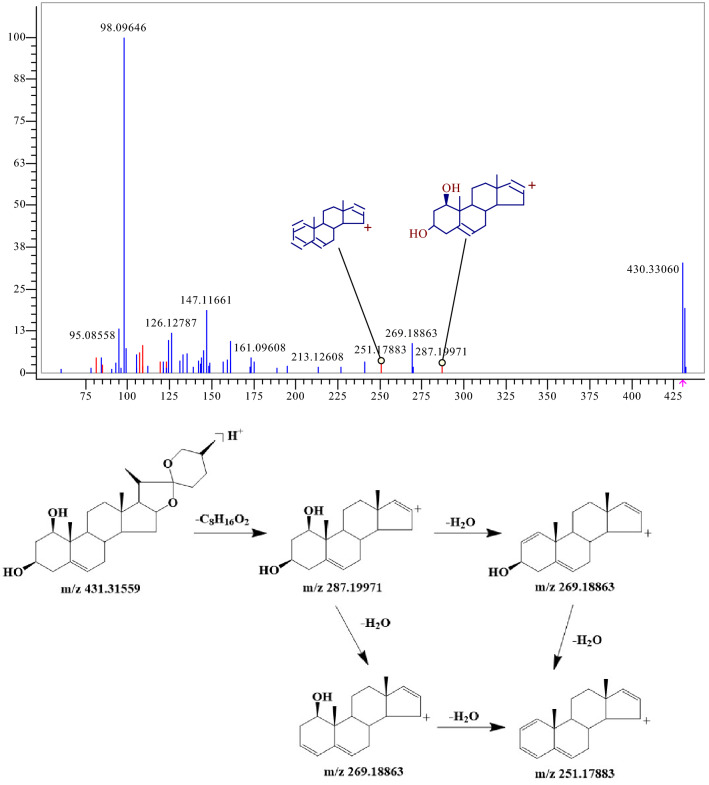
MS^2^ spectra and cracking diagram of ruscogenin.

Steroidal saponins mainly originate from OR in JSHX, the cleavage law of steroidal saponins is similar to this way. Saponins 236 possessed quasi-molecular ions [M+H] + at m/z 431.31559, and the chemical composition was C_27_H_42_O_4_. A neutral molecule C8H16O2 was lost to generate a fragment ion [M+H−C_8_H_16_O_2_] + at m/z 287.19971, a H_2_O molecule was lost in turn to generate fragment ion [M+H−C_8_H_16_O_2_−H_2_O] + at m/z 269.18863, [M+H−C_8_H_16_O_2_−2H_2_O] + at m/z 251.17883, respectively, as shown in Fig. 4, and component 236 was further identified as ruscogenin by reference standard and literature. Similarly, components 216, 220, and 221 were confirmed as Ophiopogonin D, Ophiopogonin D′, and Ophiopogonin B, respectively. According to the above rules, 52 saponins were identified, including components 1, 72, 90, 109, 110, 121, 126, 127, 129, 134, 148, 156, 162, 166, 172, 180, 187, 189, 190, 191, 192, 194, 195, 197, 198, 199, 200, 202, 206, 208, 209, 212, 214, 216, 220, 221, 223, 224, 225, 234, 235, 236, 239, 242, 243, 245, 248, 250, 259, 263, 265, 266.

#### Alkaloids

Alkaloids mainly originate from FTB in JSHX, which are generally found in nature as organic components with complex ring structures including nitrogen, and some alkaloid nitrogen can also be found outside the ring. Alkaloid components usually lose one molecule of H_2_O or CH_3_ during molecular cleavage, or appear as molecular ion peaks^24,25^.

**Figure 5:**
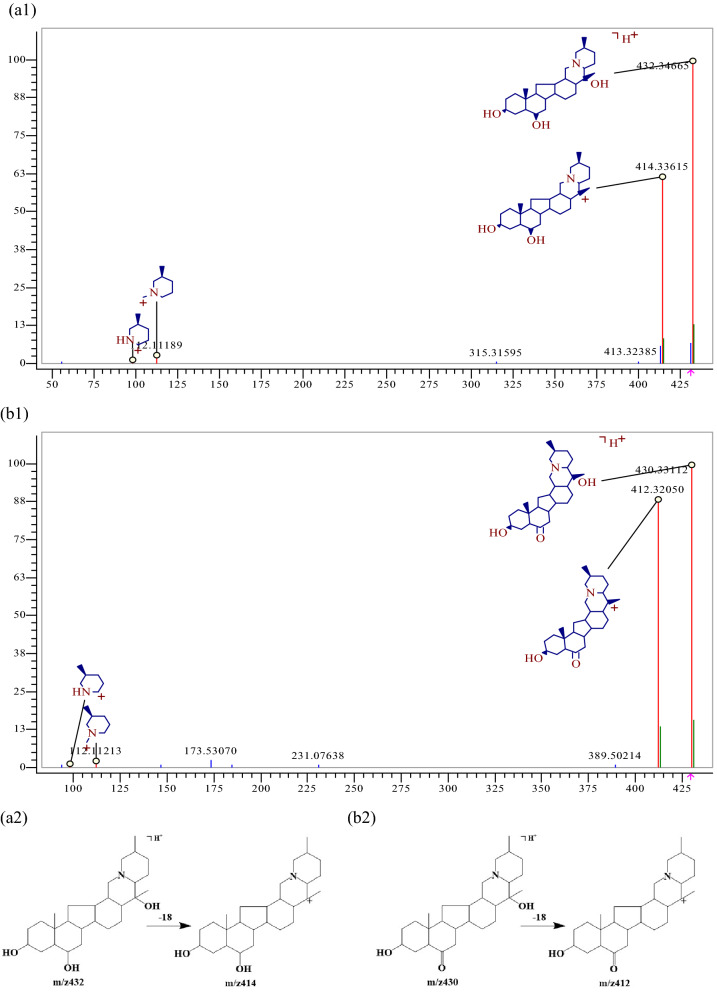
MS^2^ spectra and the cracking diagram of (**a**) Peimine, (**b**) peiminine.

For example, the ion peak [M+H] + of component 53 had an m/z of 432.34736 and its chemical composition was C_27_H_43_NO_3_. A molecule H_2_O was lost to generate a fragment ion [M+H−H_2_O] + at m/z 414.33621, as shown in Fig. 5, and component 53 was further identified as Peimine by reference standard. Similarly, the ion peak [M+H] + of component 54 had an m/z of 430.33158 and its chemical composition was calculated as C_27_H_43_NO_3_. One molecule H_2_O was lost to generate a fragment ion [M+H−H_2_O] + at m/z 412.32117, and component 54 was identified as peiminine, as shown in Fig. 5. Similarly, 31 alkaloids were identified, which were components 9, 13, 14, 16, 18, 19, 20, 22, 24, 26, 27, 28, 29, 34, 41, 44, 53, 54, 55, 60, 64, 75, 85, 108, 117, 124, 133, 149, 231, 247, 261.

#### Coumarins and terpenes

Coumarins is a lactone formed by intramolecular dehydration of CIS hydroxycinnamic acid. The basic rule of coumarins cracking is to remove several CO molecules successively, thus forming a series of [M+H–CO] + fragment peaks. Specific fragment may remove CH_3_, H_2_O, CO, CO_2_, etc., which is affected by the type and number of substituents. For example, the molecular ions peak of component 116 was m/z 313.03534 [M−H]−, and the chemical composition was C_16_H_10_O_7_. During cleavage, a molecular methyl group was lost to generate fragment ion [M−H−CH_3_]− at m/z 298.01203, a CO molecule was lost in turn to generate fragment ion [M−H−CH_3_−CO]− at m/z 270.01692, [M−H−CH_3_−2CO]− at m/z 242.02215, respectively, the component 116 could be identified as wedelolactone. In the same way, the other coumarins were identified, and totally 10 coumarins were identified as components 38, 67, 71, 116, 118, 139, 150, 161, 179 and 233.

**Figure 6:**
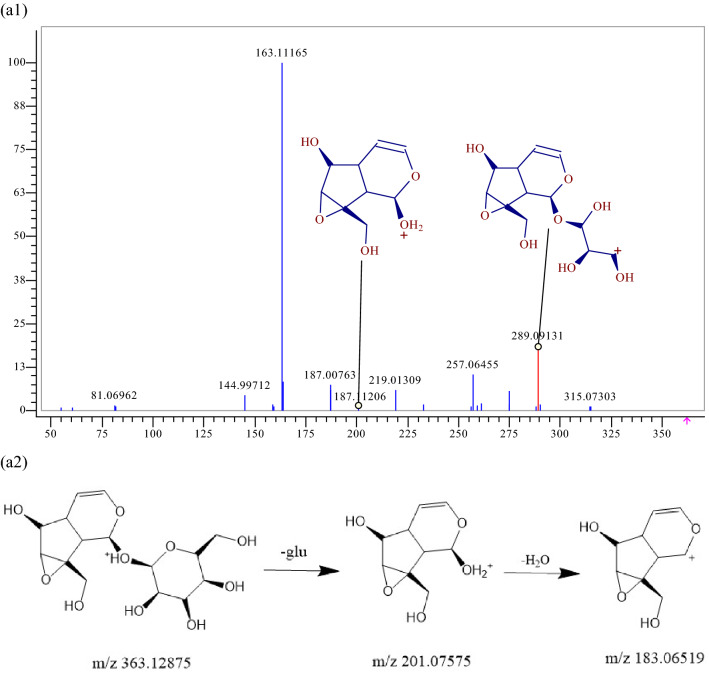
MS^2^ spectra and the cracking diagram of catalpol.

Terpenoids mainly originate from RRP and GR in JSHX. A total of 12 terpenoids were identified, including 2 iridoids, 6 terpene lactones and 4 triterpenes. RDA cleavage of terpenoids often occurs, accompanied by rearrangement during cleavage, which is generally dominated by McMelloy rearrangement, and the cleavage mode is different, usually dehydration of hydroxymethyl. Iridoids 32 possessed quasi-molecular ions [M+H] + at m/z 363.12875, and the chemical composition is C_15_H_22_O_10_. A glucosyl group is lost, generating a fragment ion [M+H−glu] + at m/z 201.07575. A H_2_O molecule was lost to generate a fragment ion [M+H−glu−H_2_O] + at m/z 183.06519, as shown in Fig. 6, and component 32 was further identified as catalpol by reference standard. Similarly, 12 terpenoids were identified: components 12, 32, 56, 57, 82, 89, 157, 188, 218, 219, 255, and 256.

#### Other components

Other components of JSHX mainly include quinones, esters, organic acids, etc., which were identified using Compound Discoverer software integrated with ChemSpider, MzCloud and other databases, comparison of reference substances and reference literature. We identified 105 other components: 23 organic acids (components 7, 10, 23, 33, 40, 61, 78, 84, 93, 98, 193, 201, 211, 217, 222, 226, 229, 230, 232, 238, 258, 260, 262), 16 esters (components 8, 31, 39, 46, 68, 101, 158, 168, 181, 205, 227, 228, 237, 249, 253, 254), 14 phenols (components 4, 30, 36, 42, 95, 144, 147, 160, 183, 196, 204, 240, 257, 264), 9 quinones (components 87, 100, 113, 120, 122, 145, 178, 186, 251), 9 phenolic acids (components 5, 15, 17, 25, 45, 49, 86, 106 and 163), 8 ketones (components 6, 92, 94, 130, 132, 169, 174, 215), 11 alcohols (components 47, 62, 103, 63, 175, 182, 207, 241, 244, 246, 252), 7 phenylpropanoids (components 11, 21, 48, 70, 91, 119, 128), 3 amides (components 99, 105, 213), 2 glycosides (components 102, 210), 1 ether (component 184), 1 amino acid (component 2) and 1 nucleotide (component 3).

### Network pharmacology analysis

#### Target prediction and PPI analysis

**Figure 7:**
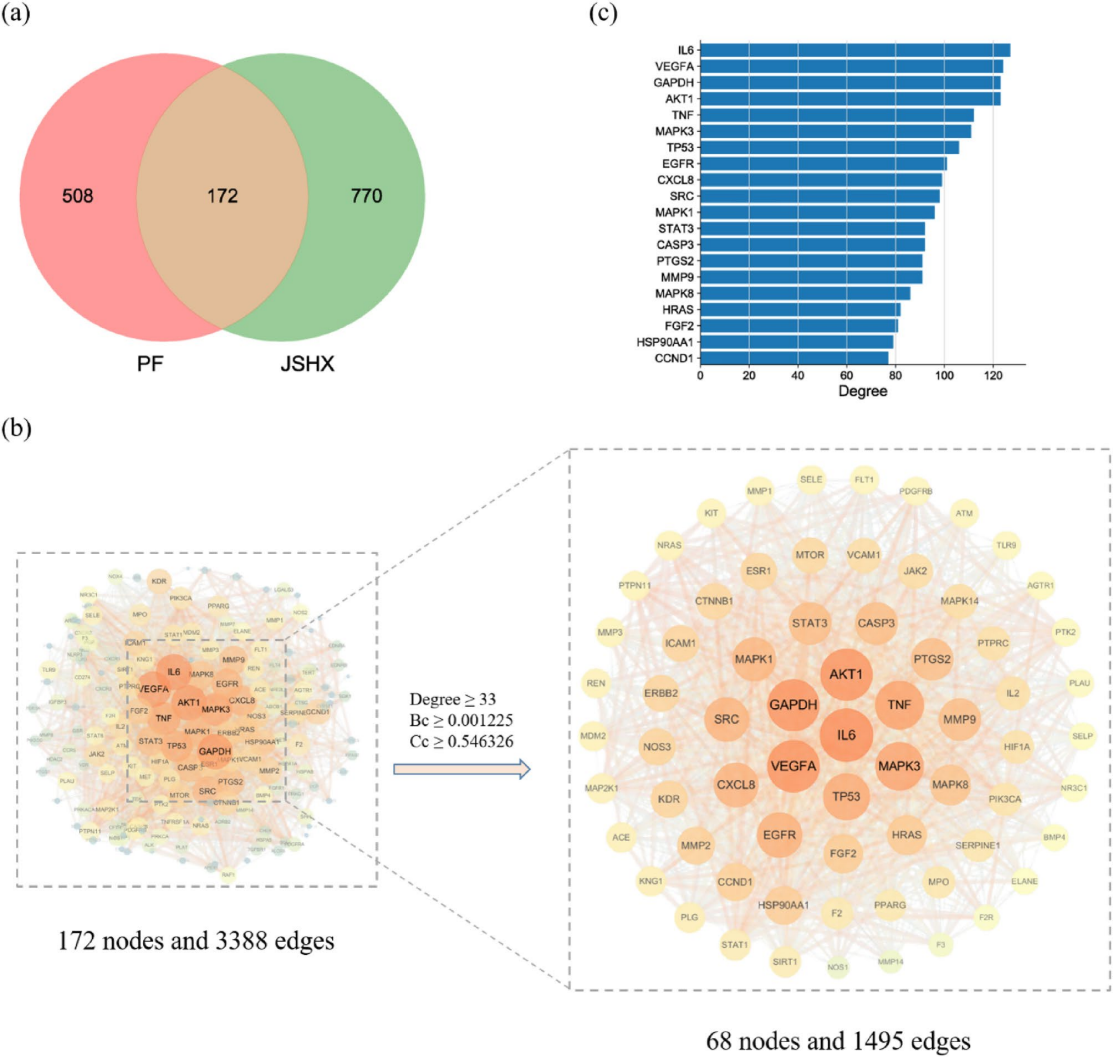
(**a**) Intersection of Venn diagram. (**b**) The process of topological screening for the protein–protein interaction network. (**c**) Node degree value. The horizontal axis represents the degree value of nodes; the vertical axis represents gene.

After removing non-target components and repeated targets, we obtained 138 components acting on 942 targets (Supplementary Table S2). In addition, 5440 PF-related genes were collected from the GeneCards database (Supplementary Table S2). To ensure the precision of target collection, based on the parameter of “Relevance score,” the index above the third median value was selected as the key index, and 680 PF-related targets were obtained. After taking the intersection of 942 predicted targets and 680 PF-related targets, 172 targets were obtained which means that they may be the key targets of JSHX in PF treatment (Fig. 7), and 90 identified components corresponding to 172 targets were found through reverse search (Supplementary Table S2).

After inputting these common targets into the STRING database, we obtained a PPI network comprising 172 nodes and 3388 edges. Using the three parameters degree, Bc, and Cc, the index above the median value was selected as the key index, the threshold value of screening was degree ≥ 33, Bc ≥ 0.001225, and Cc ≥ 0.546326, 68 hub targets were obtained (Fig. 7). Nodes in the top 20 degree were selected (Fig. 7), the results showed that Interleukin- 6 (IL6), vascular endothelial growth factor A (VEGFA), glyceraldehyde-3-phosphate dehydrogenase (GAPDH), AKT Serine/Threonine Kinase 1 (AKT1), Tumor necrosis factor (TNF) were the most vital targets of the PPI network, which may be the key targets of JSHX in PF treatment.

#### GO and KEGG pathway enrichment analysis

By using the Metascape database, 2625 GO terms and 167 KEGG pathways with P ≤ 0.01 were enriched (Supplementary Table S2).

**Figure 8:**
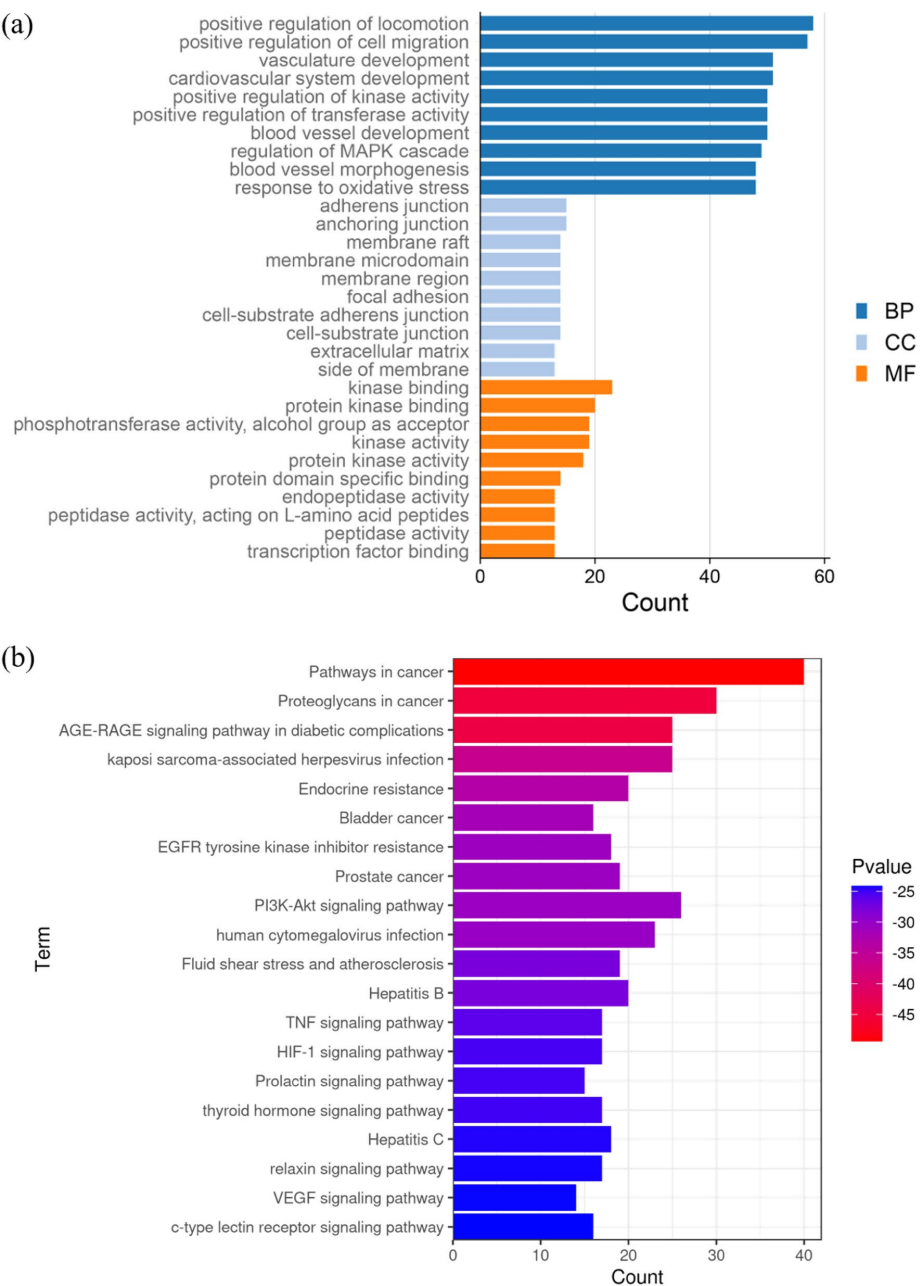
(**a**) GO enrichment results (**b**) KEGG pathway enrichment results (this figure was drawn by the Bioinformatics Data analysis and Visualization online platform (http://www.bioinformatics.com.cn/)].

Gene ontology enrichment analysis consisted of three parts: biological process (BP), molecular function (MF), and cellular component (CC). The top 20 terms for BP, CC and MF are shown as a bar plot in Fig. 8, the height of the bar represents the count, which means the number of genes observed in the category. The enrichment results showed that 2466 enrichment terms are related to BP, which cover positive regulation of cell migration, response to oxidative stress, positive regulation of kinase activity, regulation of MAPK cascade, and positive regulation of transferase activity, etc. In addition, 96 enrichment results were related to MF, including protein kinase binding, peptidase activity, and transcription factor binding, etc. A total of 63 CC items were also obtained, and the most enriched terms included membrane microdomain, side of membrane, extracellular matrix and perinuclear region of cytoplasm, etc.

The top 20 enriched KEGG pathways presented in Fig. 8 were crucially involved in the pathological process of PF. The length and color of the bar in the histogram were decided by the number of associated genes and the *p*-values. As shown in Fig. 8, the hub genes were highly related to human disease for cancer, infectious diseases, metabolic diseases, immune system and signal transduction, including the pathways in cancer, AGE-RAGE signaling pathway in diabetic complications, endocrine resistance, PI3K-Akt signaling pathway, HIF-1 signaling pathway, TNF signaling pathway, VEGF signaling pathway, and relaxin signaling pathway, etc.

#### Component-target-pathway network construction and analysis

**Figure 9:**
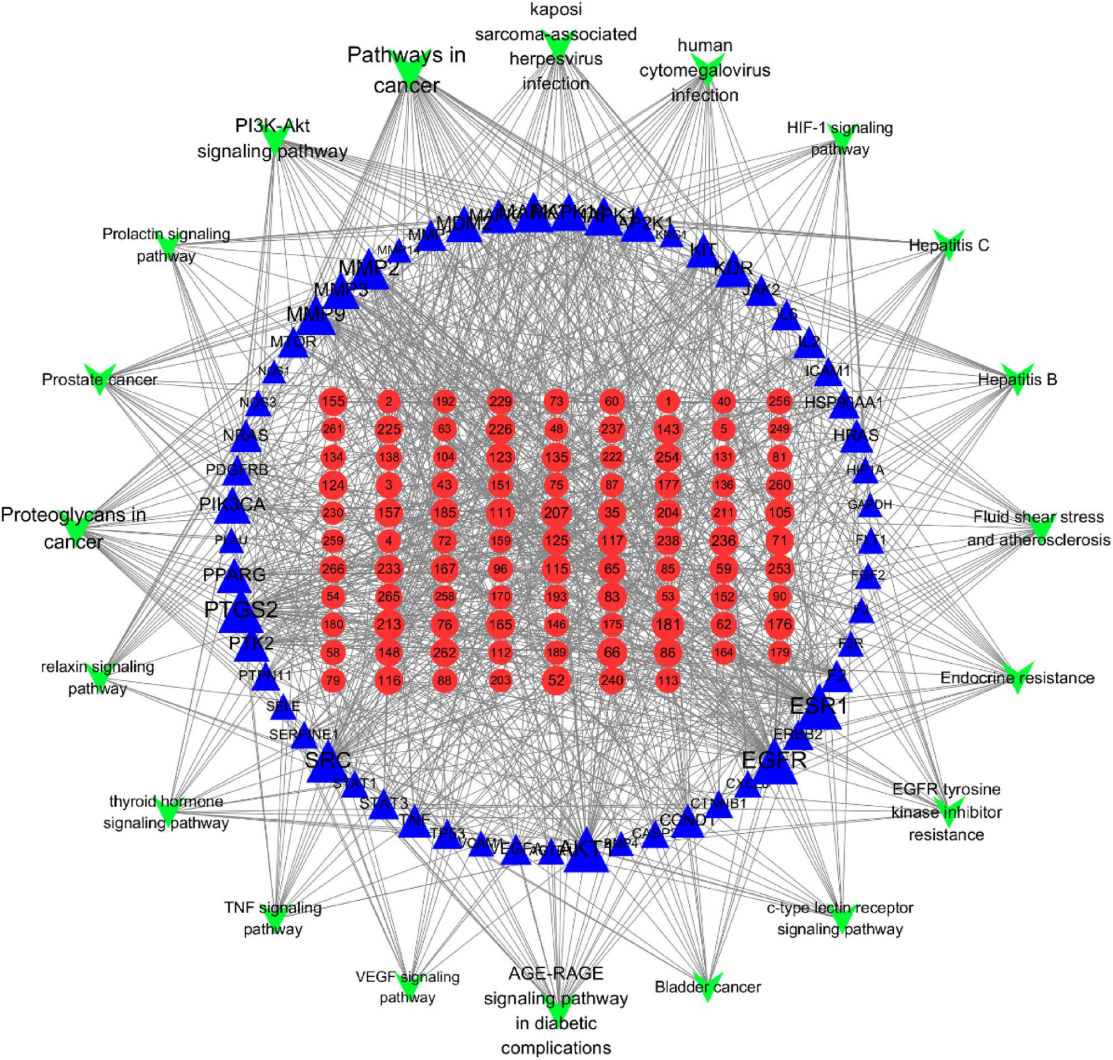
Component-target-pathway network comprised 190 nodes and 1042 edges. The red circular nodes represent 97 identified components of JSHX, the blue triangle nodes represent 57 PF-related targets, and the green V-shaped nodes represent the top 20 enriched KEGG pathways, and the node size is related to the degree value, the larger the degree value, the larger the node. The edges represent interactions between components, targets, and pathways [this figure was drawn by the Cytoscape (version 3.7.2)].

A network of validated components, targets and top 20 enriched KEGG pathways was constructed and shown in Fig. 9. The network comprised 174 nodes and 1001 edges (29). The red nodes represent 97 identified components of JSHX, the blue nodes represent 57 PF-related targets, and the green nodes represent 20 enriched KEGG pathways. This network showed that one pathway could act on multiple targets, and that one target could relate to multiple ingredients. The topological parameters degree ≥ 9.5, BC ≥ 0.004764, and CC ≥ 0.385301 were used as screening criteria, and 76 hub nodes were obtained, including 32 hub targets and 24 hub components. The top 15 targets and components are shown in Tables 1 and 2. Among the components, 3-O-methylfunicone (OMF), panaxytriol, brevisamide, 3,3′,4′,5,6,7,8-heptamethoxyflavone, ruscogenin, liquiritin, formononetin, luteolin, kaempferol, and hesperetin showed strong interactions with 10 or more PF targets, and might be the bioactive constituents of JSHX against PF.

**Table 1:** Topological properties of hub components in fifteen degree.

ID	components	Bc	Cc	Degree
181	3-O-methylfunicone	0.013633	0.408983	17
207	Panaxytriol	0.009516	0.405152	14
213	Brevisamide	0.006643	0.399538	14
66	Formononetin	0.008537	0.427160	13
176	3,3′,4′,5,6,7,8-heptamethoxyflavone	0.006516	0.414868	13
52	Liquiritin	0.005775	0.395881	12
236	Ruscogenin	0.009714	0.418886	12
111	Luteolin	0.005366	0.420925	11
123	Kaempferol	0.005366	0.420925	11
115	Hesperetin	0.011154	0.397701	11
59	Naringenin	0.010749	0.403263	11
83	Chryseriol	0.005366	0.420925	11
226	α-Linolenic acid	0.006616	0.412888	11
253	Linolenic acid ethyl ester	0.010318	0.412888	11
165	5,6,7,8,3′,4′,5′-heptamethoxyflavone	0.004787	0.405152	11

**Table 2:** Topological properties of hub genes.

Gene	Bc	Cc	Degree	Gene	Bc	Cc	Degree
EGFR	0.08644	0.461333	45	MAPK1	0.025247	0.431421	31
ESR1	0.096464	0.442455	43	MAPK14	0.021202	0.422983	28
AKT1	0.068732	0.458886	43	MMP3	0.022551	0.408983	27
PTGS2	0.113809	0.44473	42	PIK3CA	0.016302	0.422983	27
SRC	0.041904	0.440204	38	KDR	0.014933	0.412888	26
MMP2	0.051467	0.437975	35	MDM2	0.027238	0.408983	26
MMP9	0.028719	0.433584	33	PTK2	0.012452	0.408983	25
MAPK3	0.030076	0.433584	32				

### Results of molecular docking

**Figure 10:**
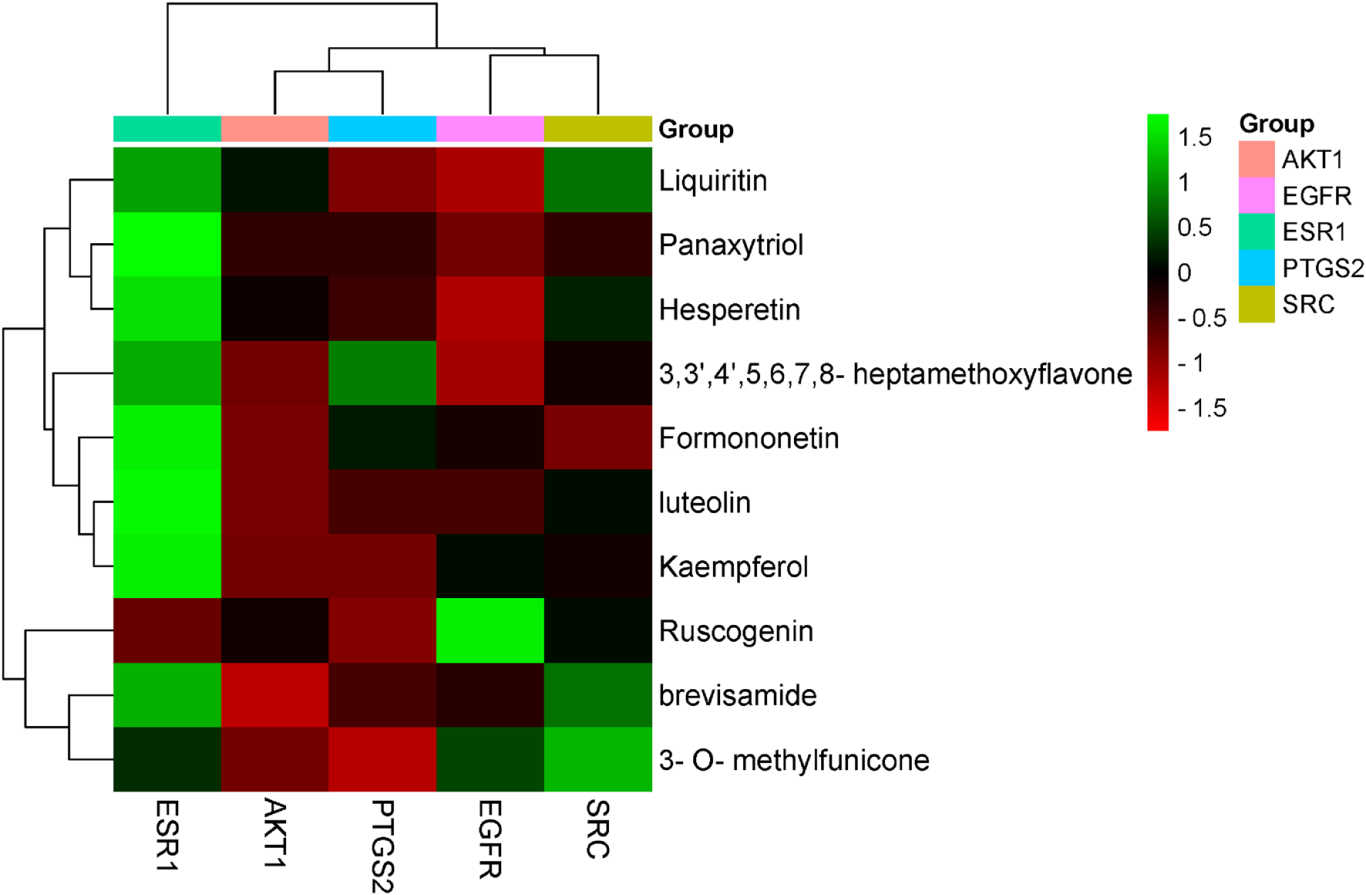
The binding energy of the core components with core targets [this figure was drawn by the Bioinformatics Data analysis and Visualization online platform (http://www.bioinformatics.com.cn/)].

The top 10 core components with corresponding five core targets were simulated by molecular docking, and the docking results were analyzed. The basic information about ligands and proteins is shown in Table 3. The results of molecular docking analysis showed that the binding free energy (ΔG in kcal/mol) of components for binding to core targets was negative (Table 4), indicating that the ligand molecules could spontaneously bind to receptor proteins. Furthermore, the binding energy was less than − 5.0 kJ/mol, which further proved the strong binding ability. The binding energies of the core components and targets are shown in Fig. 10.

**Table 3:** The basic information of ligand and protein.

Targets	PDB ID	Ligand ID	Ligand name
EGFR	5GNK	80U	1-[(3R)-3-[4-azanyl-3-[3-chloranyl-4-[(1-methylimidazol-2-yl)methoxy]phenyl]pyrazolo[3,4-d]pyrimidin-1-yl]piperidin-1-yl]prop-2-en-1-one
ESR1	3OS9	KN1	4-[1-allyl-7-(trifluoromethyl)-1H-indazol-3-yl]benzene-1,3-diol
AKT1	2F7Z	6EA	(1S)-1-(1H-INDOL-3-YLMETHYL)-2-(2-PYRIDIN-4-YL-[1,7]NAPHTYRIDIN-5-YLOXY)-EHYLAMINE
PTGS2	4COX	IMN	INDOMETHACIN
SRC	3SVV	VSP	N-(3-{[4-amino-1-(propan-2-yl)-1H-pyrazolo[3,4-d]pyrimidin-3-yl]methyl}phenyl)ethanesulfonamide

**Table 4:** The binding energy of the core components and targets.

Components	Affinity (kcal/mol)				
	EGFR	ESR1	AKT1	PTGS2	SRC
3-O-methylfunicone	− 6.8	− 6.9	− 7.6	− 7.9	− 6.3
Panaxytriol	− 6.1	− 5.0	− 5.9	− 5.9	− 5.9
Bbrevisamide	− 7.7	− 7.0	− 8.2	− 7.8	− 7.2
3,3′,4′,5,6,7,8-heptamethoxyflavone	− 7.8	− 7.1	− 7.7	− 7.2	− 7.5
Ruscogenin	− 6.5	− 11.2	− 10.0	− 11.5	− 9.6
Liquiritin	− 9.0	− 8.3	− 8.6	− 8.9	− 8.4
Formononetin	− 8.5	− 7.4	− 8.9	− 8.3	− 8.9
Luteolin	− 8.9	− 7.7	− 9.1	− 8.9	− 8.6
Kaempferol	− 8.2	− 7.5	− 8.6	− 8.6	− 8.3
Hesperetin	− 9.2	− 7.5	− 8.5	− 8.7	− 8.3

**Figure 11:**
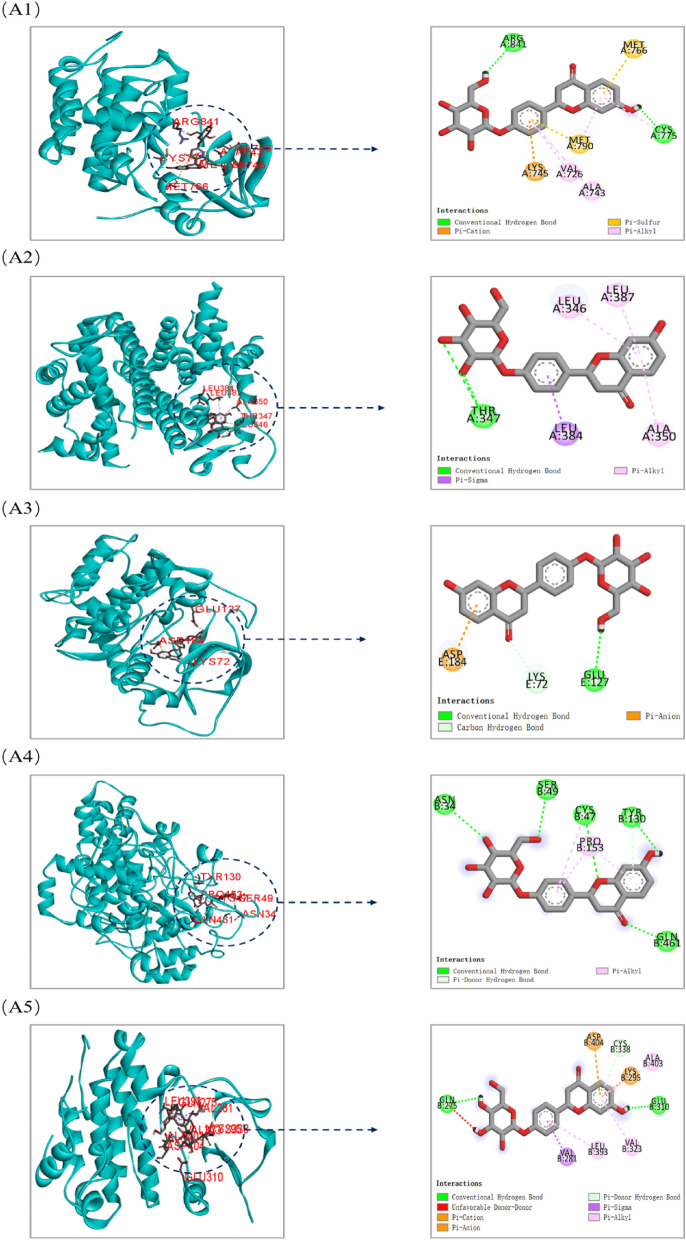

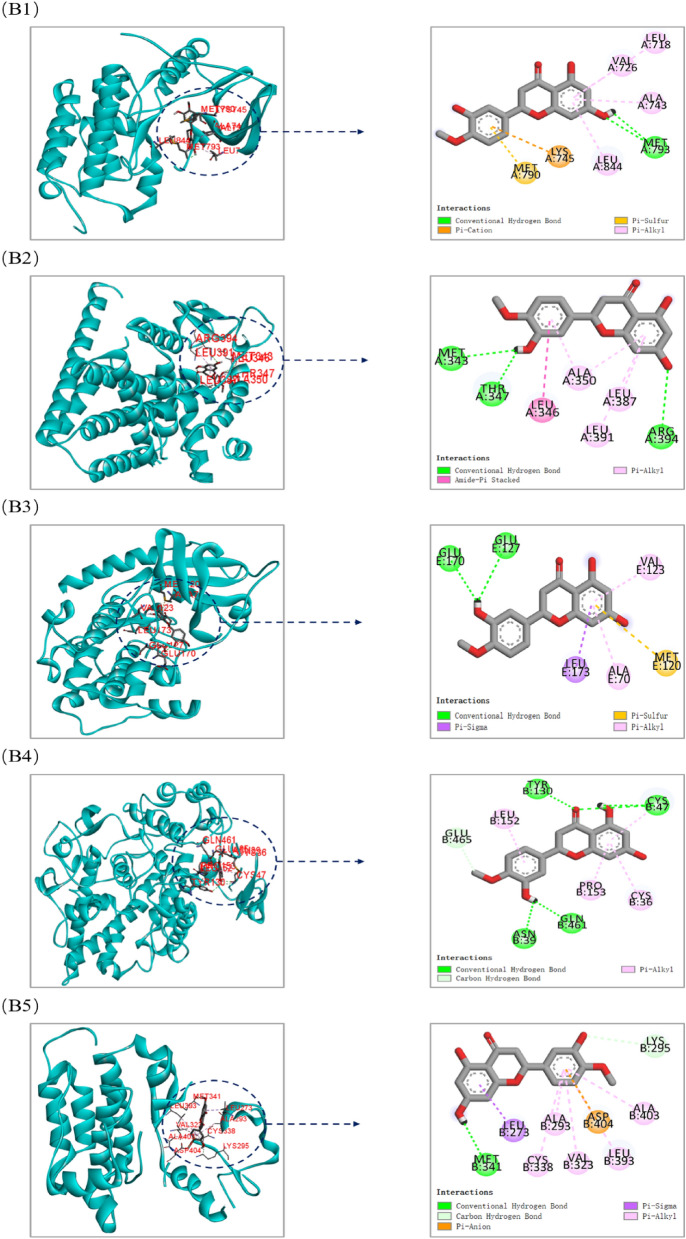
Molecular docking models of liquiritin and hesperetin binding to the five core targets. (**A1**–**A5**) the binding interaction between liquiritin and EGFR, ESR1, AKT1, PTGS2, SRC, respectively. (**B1**–**B5**) the binding interaction between hesperetin and EGFR, ESR1, AKT1, PTGS2, SRC, respectively (this figure was drawn by the Discovery Studio Client software (version 4.5)].

Both liquiritin and hesperetin had strong binding ability with five core targets. Visualization of the docking results was performed using Discovery Studio 4.5 Client software (Fig. 11). Liquiritin formed hydrogen bonds with ABG841 and CYS775 residues on EGFR; THR347 residues on ESR1; GLU127 residues on AKT1; ASN34, SER49, CYS47, TYR130 and GLN461 residues on PTGS2; GLU310 and GLN275 residues on SRC. Hesperetin formed hydrogen bonds with MET793 residues on EGFR; MET343, THR347 and ARG394 residues on ESR1; GLU170 and GLU127 residues on AKT1; TYR130, CYS47, ASN39 and GLN461 residues on PTGS2; and MET341 residues on SRC.

### Experimental validation

Studies have shown alveolar epithelial cells A549 are one of the mediators for PF detection. Transforming growth factor β1 (TGF β1) promotes pulmonary fibrosis, which mediates epithelial mesenchymal transformation of human alveolar epithelial cells, resulting in extracellular matrix deposition^26^. It was found that human lung cancer A549 cells were type II alveolar epithelial cells, which became a common in vitro model to study the mechanism of pulmonary fibrosis after induction with TGF-β1^27^. More and more evidences indicate that autophagy, which maintains intracellular homeostasis, is closely related to the occurrence and development of pulmonary fibrosis^28^. Therefore, we selected TGF-β1 induced A549 cells as the in vitro model. According to the network pharmacology and molecular docking analysis, three bioactive components including ruscogenin, liquiritin, hesperetin had the better molecular docking results. To further evaluate the results obtained by systematic pharmacologic analyses, these components were selected from JSHX to examine the potential effects of anti-inflammatory and inhibiting the differentiation of fibroblasts by using TGF-β1 (7.5 ng/ml)-stimulated A549 cells. We employed ELISA assay for E-cad, α-SMA and FN to confirm the predicted anti-PF effects of three bioactive components.

**Figure 12:**
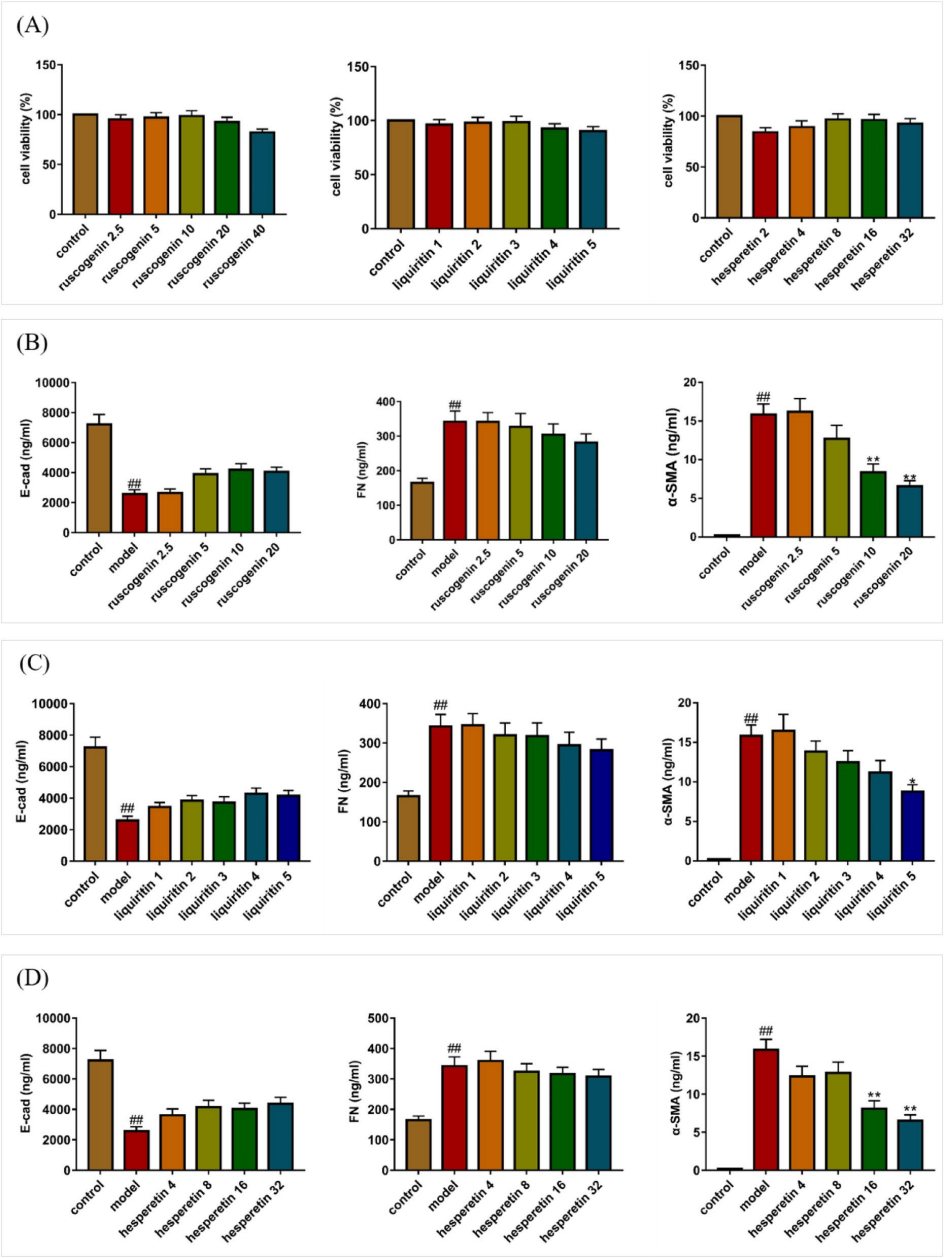
Effect of ruscogenin, liquiritin, hesperetin on A549 cells. (**A**) The effect of ruscogenin, liquiritin, hesperetin on A549 cell viability were detected by MTT analysis. (**B**–**D**) The effects of different concentrations of ruscogenin, liquiritin, hesperetin on the contents of E-cad, FN and α-SMA in pulmonary fibrosis model were determined by ELISA. Values represented as mean ± SD. ^##^P < 0.01 versus control group. **P < 0.01, *P < 0.05, versus model group [this figure was drawn by the GraphPad Prism software (version 7.00)].

Firstly, we determined the effects of different doses of ruscogenin, liquiritin, hesperetin on the viability of A549 cells using MTT assay. As shown in Fig. 12, ruscogenin (2.5, 5, 10, 20 μg/ml), liquiritin (1, 2, 3, 4, 5 μg/ml), hesperetin (4, 8, 16, 32 μg/ml) had high cell viability (> 85%). Therefore, these concentrations were selected for subsequent experiments. As shown in Table 5, compared with the control group, the contents of α-SMA and FN in TGF-β1-induced cells increased significantly, and the content of E-cad decreased significantly. With different concentrations of ruscogenin, liquiritin, hesperetin treatment, the content of α-SMA and FN decreased to varying degrees, the content of E-cad increased when compared with the model group. As shown in Fig. 12, regarding the α-SMA content, each dose group showed a significant downward trend; For FN, only the high-dose group showed a significant downward trend; Similarly, the E-cad content of high dose group showed a rising trend.

**Table 5:** The effect of ruscogenin, liquiritin, hesperetin on A549 cells (values represented as mean ± SD. ^##^P < 0.01 versus control group. **P < 0.01, *P < 0.05, versus model group).

Groups	Concentrations/(mg/ml)	E-cad/(ng/ml)	α-SMA/(ng/ml)	FN/(ng/ml)
Control	0.0	7215.4	0.21	164,832.3
Model	0.0	2578.3^##^	15.84^##^	341,945.2^##^
Ruscogenin	2.5	2643.9	16.19	341,306.5
	5.0	3893.4	12.71	326,684.1
	10.0	4195.4	8.38**	303,773.8
	20.0	4051.3	6.58**	281,629.9
Liquiritin	1.0	3439.3	16.46	345,307.5
	2.0	3833.7	13.82	319,617.8
	3.0	3724.3	12.48	317,404.5
	4.0	4277.7	11.18	294,040.3
	5.0	4152.2	8.78*	281,506.8
Hesperetin	4.0	3610.8	12.34	359,093.6
	8.0	4148.4	12.79	323,710.7
	16.0	4028.9	8.09**	316,028.8
	32.0	4367.9	6.53**	307,689.5

## Discussion

In this study, we analyzed the chemical components of JSHX and explore its molecular mechanisms against PF using integrative strategy, including chemical components analysis, network pharmacology prediction, molecular docking, and experiment in vitro. Firstly, the total ion chromatograms of JSHX were obtained by UPLC-Orbitrap Fusion MS. Different mobile phase systems (methanol–water, acetonitrile–water, acetonitrile-0.1% formic acid water, methanol-0.1% formic acid water) and different elution gradients were investigated to optimize reliable and effective chromatography conditions. In addition, due to the different response modes of various components in the formula, positive and negative ion-scanning modes were selected for simultaneous detection line monitoring. A total of 266 chemical constituents (193 in ESI+ and 73 in ESI−) were identified or tentatively characterized by matching the MS raw data to mzCloud, Chemspider and other built-in databases using the Compound Discoverer 3.0 software, including 56 flavonoids, 52 saponins, 31 alkaloids, 10 coumarins, 12 terpenoids and 105 other components, which could be concluded that the developed UPLC-Orbitrap Fusion MS method was sensitive for identifying the chemical components in JSHX, and that the result of the study provided more chemical material basis information for further research of JSHX.

Network pharmacology can reveal the relationships between targets/pathways and chemical components, and establish a “component-target-pathway” network to clarify the molecular mechanisms of drug therapy for disease. In the present study, 90 of the 266 identified components of JSHX were considered to have potential key anti-PF effects by acting on 172 related targets. To explore the key bioactive components and underlying mechanisms of JSHX against PF, a multidimensional component-target-pathway network was further constructed and visualized using Cytoscape 3.7.2. Twenty-four key bioactive components were screened based on three topological parameters, which may improve the pathological status of patients with PF by regulating fifty-seven corresponding targets, including twelve flavonoids, one terpenoid, one saponin, one coumarin, one alkaloid, and six other components, which proved that JSHX had multicomponent synergistic effect in the treatment of PF. Studies have shown that TGF-β can promote the proliferation of lung fibroblasts and collagen synthesis, which is the key cytokine leading to fibrosis, so we chose TGF-β as PF inducer^29,30^. As the predicted components, ruscogenin, liquiritin and hesperetin exerted good anti-PF effects based on TGF-β-stimulated A549 cells.

Among these bioactive components, flavonoids have anti-inflammatory and antioxidant activities, which are more consistent with PF treatment^31,32^. Luteolin, and kaempferol were reported to exert therapeutic effects on PF by inhibiting the proliferation and inflammation of the lung fibroblasts in rat^33,34^. Hesperetin play a protective role in PF, which can inhibit inflammatory response by reducing the levels of TGF-β1, TNF-α, and IL-1β^35^. In addition, OMF, panaxytriol, ruscogenin, and brevisamide have relatively high degree values, and can be used as the main active components of JSHX against PF. Among them, ruscogenin, the main saponins in JSHX, can significantly inhibit TGF-β1-induced increase of FN and COL-I in PF model cell^17^. OMF is a secondary metabolite produced by penicillium pinophilum, which can induce the apoptotic pathway and inhibit cell motility and so may have potential as a naturally derived antitumor drug^36^. Panaxytriol shows potential anti-inflammatory activity by inhibiting the mRNA expression of proinflammatory cytokines such as TNF-α, IL-1b, and IL-6^37^. In addition, Panaxytriol can inhibit the proliferation of lung cancer cells and induce apoptosis by downregulating ERK1/2 and mTORC1 pathways^38^. Studies have shown that brevisamide can improve microcirculation disturbance in the lung, promote oxygen utilization, eliminate oxygen free radicals, resist oxidation, and inhibit apoptosis^39^. The mechanisms of these active ingredients cover most aspects of the pathological process of PF, and the target information involved is basically consistent with our predicted results, indicating that the comprehensive pharmacological strategy has certain predictive accuracy. Among the predicted potential targets, EGFR, ESR1, AKT1, PTGS2, SRC, IL6, and VEGFA had high frequency, and were involved in several biological processes, including positive regulation of cell migration, positive regulation of kinase activity, response to oxidative stress, regulation of MAPK cascade, regulation of cell adhesion, which were closely related to the pathogenesis of PF.

KEGG enrichment analysis showed that the potential targets were highly related to human disease for cancer, infectious diseases, metabolic diseases, immune system and signal transduction, including the pathways in cancer, endocrine resistance, EGFR tyrosine kinase inhibitor resistance, HIF-1 signaling pathway, TNF signaling pathway, PI3K-Akt signaling pathway, and VEGFA signaling pathway, etc. Of these, the PI3K-Akt signaling pathway is the key pathway, that participates in the pathophysiological process of apoptosis, proliferation and differentiation^40^, and affects the overall process of PF by activating downstream mTOR and HIF-1α, CTGF, VEGF and other factors to regulate ROS production and metabolism, extracellular matrix deposition, lung fibroblast production, and collagen production^41–43^. PI3K is an important part of the signal transduction process of the growth factor receptor superfamily. Activated PI3K can cause downstream protein phosphorylation and promote the mesenchymal transformation and extracellular matrix deposition of alveolar epithelial cells to form pulmonary fibrosis^44^. Akt, a direct target protein downstream of PI3K, can participate in the regulation of cell proliferation and metabolism, and promote fibrosis related gene transcription and protein synthesis^45^. Activated Akt activates the downstream HIF-1α signaling pathway, inhibiting alveolar surfactant production and normal repair of alveolar epithelial cells^46^. In addition, it can also activate NF-κB to initiate downstream inflammatory responses that produce inflammatory factors such as TNF-α and IL-6, thereby exacerbating fibrosis^47^. JSHX may exert anti-PF effects by inhibiting lung tissue inflammatory responses and apoptosis through regulating the expression of related genes in the PI3K-Akt signaling pathway. The HIF-1α signaling pathway is involved in apoptosis, proliferation, metabolism, growth and transformation, membrane transport, secretion and chemotaxis, and plays an important role in the pathogenesis of inflammation, tumors, metabolism, and cardiovascular diseases^48^. HIF-1α can mediate the transformation of bronchial and alveolar epithelial stromal cells and participate in the occurrence of PF^49^. The results showed that the JSHX had a certain intervention effect on the formation of PF.

Molecular docking technology was used to verify the binding of core components and targets related with inflammation and oxidative stress. The binding free energy of components with the core targets was less than − 5.0 kJ/mol, which indicated that the ligand molecules could spontaneously bind to receptor proteins with a strong binding force. Based on the network pharmacology and molecular docking results, we test the effect of three bioactive components (ruscogenin, liquiritin and hesperetin) presented on differentiation of fibroblast in TGF-β1-induced A549 cells. Experiment in vitro verified that the bioactive components inhibited the increase of α-SMA and FN, and the decrease of E-cad, and relieved pathological manifestation of PF. The results indicated that JSHX may play a role in PF treatment through anti-inflammatory, antioxidant and suppressing differentiation of fibroblasts into myofibroblasts synergistic effects.

## Conclusions

A valid, sensible and accessible UPLC-Orbitrap Fusion MS method was established for the chemical ingredient analysis of JSHX. Based on the identified components, network pharmacology and molecule docking were used to screen the effective components of JSHX and clarify their underlying mechanisms for PF treatment, and were further validated by experiments. The inhibitory effects of 3-O-methylfunicone, panaxytriol, ruscogenin, liquiritin and hesperetin displayed on the PI3K-Akt, HIF-1 and TNF signaling pathways might be the mechanisms of action of JSHX against PF. These findings provide an experimental basis for the scientific connotation and clinical application of JSHX against PF. However, the consistency of the components predicted by network pharmacology with those that ultimately enter the organismal circulation cannot be determined, and deeper mechanisms still need to be further demonstrated by more animal experiments, which will be the focus of our future research.

## Supplementary Information

Supplementary Legends.

Supplementary Table S1.

Supplementary Table S2.

## Abbreviations

JSHX: Jinshui Huanxian granules
CM: Chinese medicine
PF: Pulmonary fibrosis
UPLC-Orbitrap Fusion MS: Ultra-high performance liquid chromatography-Orbitrap Fusion mass spectrometry
PPI: Protein–protein interaction
GO: Gene Ontology
KEGG: Kyoto Encyclopedia of Genes and Genomes
Bc: Betweenness centrality
Cc: Closeness centrality
RDA: Retro-Diels-Adel
glu: Glucosyl
BP: Biological process
MF: Molecular function
CC: Cellular component
RT: Retention time
PG: Panax ginseng C. A. Mey.
OR: Ophiopogonis Radix
RRP: Radix Rehmanniae Praeparata
TF: Trichosanthis Fructus
FTB: Fritillariae Thunbergii Bulbus
MC: Moutan cortex
EF: Epimedii folium
GS: Ginkgo semen
CRP: Citri Reticulatae Pericarpium
GR: Glycyrrhizae radix et rhizome
MAPK: Mitogen-activated protein kinase
Nf-κB: Nuclear factor kappa B
PI3K/Akt: Phosphatidylinositol-3-kinase/protein kinase B
Nrf2: Nuclear factor erythroid-2 related factor 2
ROS: Reactive oxygen species
TIC: Total ion chromatograms
IL6: Interleukin-6
VEGFA: Vascular endothelial growth factor A
GAPDH: Glyceraldehyde-3-phosphate dehydrogenase
AKT1: AKT serine/threonine kinase 1
TNF: Tumor necrosis factor
TGF-β1: Transforming growth factor β1
α-SMA: α-Smooth muscle actin
FN: Fibronectin
E-cad: E-cadherin
FBS: Fetal bovine serum
ELISA: Enzyme-linked immunosorbent assay
