# Supplementary material for: Systematic characterization of the components and molecular mechanisms of Jinshui Huanxian granules using UPLC-Orbitrap Fusion MS integrated with network pharmacology
Source: Sci Rep. 2022 Jul 21;12:12476. doi: 10.1038/s41598-022-16711-4 (PMC9304367; doi:10.1038/s41598-022-16711-4)
Supplement: Supplementary file 1 — Supplementary Legends. [file 41598_2022_16711_MOESM1_ESM.docx]

**Supplementary material**

The supplementary materials of this article are as follows:

[Supplementary Table S1](Supplementary%20table1.xlsx). The detailed information of chemical components identified in JSHX based on UPLC-Orbitrap Fusion MS.

[Supplementary Table S2](Supplementary%20table%202.xlsx). The details of network pharmacology results.
